# Disseminated intravascular coagulation after splenic artery aneurysm coils embolization: an unexpected surprise

**DOI:** 10.1093/gastro/goad025

**Published:** 2023-05-25

**Authors:** Marco Biolato, Alessandro D’Errico, Fiammetta Maria Rognoni, Giuseppe Marrone, Luca Laurenti, Alessandro Cina, Antonio Grieco

**Affiliations:** Department of Medical and Surgical Sciences, Fondazione Policlinico Universitario Agostino Gemelli IRCCS, Rome, Italy; Institute of Internal Medicine, Catholic University of the Sacred Heart, Rome, Italy; Institute of Internal Medicine, Catholic University of the Sacred Heart, Rome, Italy; Institute of Internal Medicine, Catholic University of the Sacred Heart, Rome, Italy; Department of Medical and Surgical Sciences, Fondazione Policlinico Universitario Agostino Gemelli IRCCS, Rome, Italy; Institute of Internal Medicine, Catholic University of the Sacred Heart, Rome, Italy; Department of Diagnostic imaging, Radiation oncology and Hematology, Fondazione Policlinico Universitario Agostino Gemelli IRCCS, Rome, Italy; Institute of Hematology, Catholic University of the Sacred Heart, Rome, Italy; Department of Diagnostic imaging, Radiation oncology and Hematology, Fondazione Policlinico Universitario Agostino Gemelli IRCCS, Rome, Italy; Institute of Radiology, Catholic University of the Sacred Heart, Rome, Italy; Department of Medical and Surgical Sciences, Fondazione Policlinico Universitario Agostino Gemelli IRCCS, Rome, Italy; Institute of Internal Medicine, Catholic University of the Sacred Heart, Rome, Italy

## Introduction

The Society for Vascular Surgery recommends treating non-ruptured splenic artery aneurysms (SAAs) of >3 cm with a demonstrable increase in size or with associated symptoms [1]. Percutaneous intervention with embolization coils or covered stents has become popular due to its high technical success rates and low morbidity [2, 3].

Reported complications of endovascular therapy include access-related problems and site-specific complications; in particular, reported complications of SAA coils embolization include splenic infarct, reperfusion of the aneurysm, and post-embolization syndrome [4, 5]. To the best of our knowledge, no cases of disseminated intravascular coagulation (DIC) after visceral artery aneurysm coils embolization have been reported in the literature so far. DIC is potentially fatal without prompt recognition and management.

## Case report

**Figure 1.**
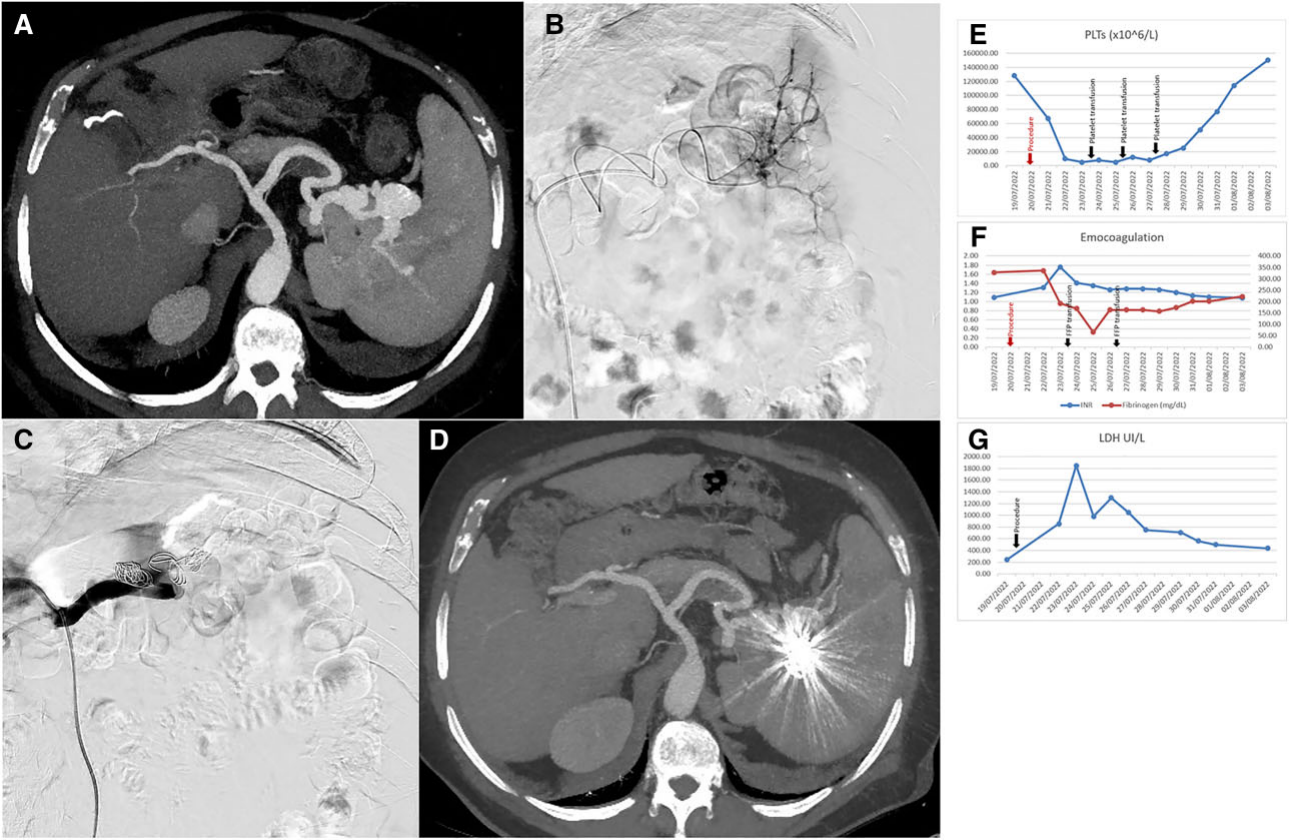
The clinical data of the case. (A) Preoperative computed tomography sub-volume axial maximum intensity projection reconstruction of arterial phase showing two saccular aneurysms of the middle (diameter 16 mm) and distal (diameter 30 mm) portion of the splenic artery. (B) Digital subtraction angiography with catheterization of the hilar portion of the splenic artery by using a coaxial microcatheter. (C) Due to the severe tortuosity of the splenic artery, the “packing” by coils of the distal aneurysm was impossible and therefore the splenic artery was occluded by coils in the middle portion. After releasing three coils and achieving slowing flow, the occlusion of the artery was performed by using a transcatheter injection of 2,500 UI of thrombin. (D) Sub-volume axial maximum intensity projection reconstruction of the arterial phase of computed tomography performed 4 days after the procedure. Coils are migrated in the distal splenic artery. In the portal phase of the exam, no relevant alterations of splenic parenchyma were detected (images not shown). (E) Platelet count (PTL) trend. Black arrows indicate platelet transfusion. (F) Coagulation parameter trend: international normalized ratio (INR) and fibrinogen. Black arrows indicate fresh frozen plasma (FFP) transfusions. (G) Lactate dehydrogenase (LDH) trend.

A 69-year-old man with a history of multiple aneurysms located in the distal splenic artery, with a diameter up to 3 cm, was admitted to our ward to undergo a scheduled procedure of embolization of his SAAs (Figure 1A). His medical history included compensated liver cirrhosis caused by hepatitis C virus, successfully treated in 2015, complicated by spontaneous splenorenal shunt and portal vein thrombosis, which was treated with warfarin and subsequently heparin until 2021 (suspended after a post-traumatic subdural hematoma); he had Class I obesity (body mass index 34.6 kg/m^2^).

At admission, the patient had no symptoms of clinical relevance. Laboratory workup reported: platelet count 128 × 10^9^/L (normal range: 150–450 × 10^9^/L in our hospital), hemoglobin 156 g/L, glutamic-oxalacetic transaminase 24 IU/L, glutamate pyruvate transaminase 13 IU/L, total bilirubin 20.5 μmol/L, creatinine 70.7 μmol/L, lactate dehydrogenase (LDH) 241 IU/L, international normalized ratio (INR) 1.09, activated partial thromboplastin time (aPTT) 34.1 s, and fibrinogen 9.6 μmol/L.

The radiological procedure plan was to close the aneurysm by detaching metal coils endovascularly; in this case, the “packing” by coils of the distal aneurysm was not technically feasible and therefore the splenic artery was occluded by coils in the middle portion; after the detachment of eight coils, there was still a detectable flow so the occlusion of the artery was completed with a transcatheter injection of 2,500 UI of thrombin (Floseal©) (Figure 1B and C).

The day after the procedure, the patient presented a reduced platelet count (67 × 10^9^/L). Lab tests 24 h later showed severe thrombocytopenia (platelet count 10 × 10^9^/L), with a decrease in fibrinogen (1.9 μmol/L), an elevation of INR (1.31), aPTT (41.1 s), raised LDH (1,675 IU/L, normal range 109–250 IU/L), and D-dimer elevation (>35, 000 ng/mL). The search for antiphospholipid antibodies, antinuclear antibody, anti-neutrophil cytoplasmatic antibody, anti-smooth muscle antibody, anti-liver kidney microsomal antibody, anti-centromere antibody, anti-nucleosomes antibody, rheumatoid factor, and the serology for hepatitis B virus, Epstein-Barr virus, cytomegalovirus, and human immunodeficiency virus were all negative. The schistocyte count was negative at the first determination and became positive after 3 days. A mild positivity of the antibodies anti-ADAMTS13 was found (18.7 U/mL, normal values < 15 U/mL), but the ADAMTS13 activity levels were normal (52.9%, normal values > 50%). Bone marrow aspiration was performed, which showed normal cell count, normal megakaryocytes, and no sign of other acute ongoing hemopathy. Abdominal computed tomography showed multiple ischemic areas in the spleen and migration of the coils inserted in the middle splenic artery with subsequent patency of the smaller and proximal aneurysm, but persistent occlusion of the larger one (Figure 1D).

Based on the hypothesis of an immune thrombocytopenia reaction, steroid therapy (prednisone 40 mg i.v. b.i.d.) was started. In the following days, however, the platelet count continued to decrease down to 5 × 10^9^/L, with a progressive increase in INR (1.76), aPTT (44.1 s), D-dimer (35 ,200 ng/mL), and LDH (1,296 IU/L), and no change in the fibrinogen level (1.9 μmol/L). Multiple platelet and plasma transfusions were performed. The patient started to develop multiple spontaneous skin hematomas, with a progressive decrease in the hemoglobin level, which went down to 93 g/L. The patient had no other significant symptoms. Main laboratory data trend and transfusions performed are shown in Figure 1E–G.

A week later, the platelet count and the coagulation parameters started to slowly recover. Steroid therapy was progressively tapered off, without relapse. The patient was discharged 14 days after the procedure; the platelet count was 150 × 10^9^/L and the coagulation parameters were back within normal ranges.

Considering laboratory test abnormalities, imaging findings, and steroid-resistant thrombocytopenia, a final diagnosis of DIC was made (International Society on Thrombosis and Haemostasis score of 6 points).

## Discussion

We reported a case of a patient who had developed DIC after SAAs coils embolization together with the administration of a lyophilized thrombin powder; the intravascular administration of thrombin by ultrasound-guided injection is commonly used as a treatment for pseudoaneurysms of the femoral artery secondary to percutaneous procedures [6] and, in recent years, for the management of visceral aneurysms [7, 8].

The patient had no other apparent triggers, so the provocative event for DIC is confidently attributed to the procedure performed the day before. Keeping in mind that the coils were not removed and the acute event resolved itself only with supportive measures, we hypothesize that the triggering cause was the intravenous administration of the thrombin-based hemostatic agent.

In this case, we decided not to administer unfractionated heparin even though the patient was experiencing DIC with an ongoing thrombotic event; the decision not to treat was based on the hemodynamic stability of the patient and the very low platelet count requiring multiple transfusions.

We do not know whether there is a dose–effect correlation between the use of thrombin and the development of DIC. There are no established thrombin dose recommendations for the treatment of large aneurysms combined with coils. In the literature, a dose of 1,000 UI was reported for percutaneous embolization of a visceral pseudoaneurysm that was 2 cm in diameter [9] and up to 3,000 UI by transcatheter administration [10]. In our case, the overall dose of 2,500 UI of thrombin was reached by sequential multiple injection of 2 mL of thrombin solution (500 UI) followed by angiographic control, until the occlusion of the aneurysm was achieved.

We believe that both clinicians and interventional radiologists should keep in mind this possible severe side effect after an interventional procedure like ours in order to promptly start the appropriate treatments.

## Authors’ Contributions

M.B. wrote the paper; A.D.E. and F.M.R. collected data and prepared the figure; G.M., L.L., A.C., and A.G. revised the paper for important intellectual content. All authors have read and approved the final version of the manuscript.
